# Health behaviour change interventions for couples: A systematic review

**DOI:** 10.1111/bjhp.12227

**Published:** 2017-02-02

**Authors:** Emily Arden‐Close, Nuala McGrath

**Affiliations:** ^1^ Department of PsychologyFaculty of Science and TechnologyResearch Centre for Behaviour ChangeBournemouth UniversityPooleUK; ^2^ Department of Primary Care and Population SciencesUniversity of SouthamptonUK; ^3^ Department of Social Statistics and DemographyUniversity of SouthamptonUK; ^4^ Africa Centre for Health and Population StudiesUniversity of KwaZulu NatalDurbanSouth Africa

**Keywords:** couples, health behaviour change, review, interventions

## Abstract

**Objectives:**

Partners are a significant influence on individuals’ health, and concordance in health behaviours increases over time in couples. Several theories suggest that couple‐focused interventions for health behaviour change may therefore be more effective than individual interventions.

**Design:**

A systematic review of health behaviour change interventions for couples was conducted.

**Methods:**

Systematic search methods identified randomized controlled trials (RCTs) and non‐randomized interventions of health behaviour change for couples with at least one member at risk of a chronic physical illness, published from 1990–2014.

**Results:**

We identified 14 studies, targeting the following health behaviours: cancer prevention (6), obesity (1), diet (2), smoking in pregnancy (2), physical activity (1) and multiple health behaviours (2). In four out of seven trials couple‐focused interventions were more effective than usual care. Of four RCTs comparing a couple‐focused intervention to an individual intervention, two found that the couple‐focused intervention was more effective.

**Conclusions:**

The studies were heterogeneous, and included participants at risk of a variety of illnesses. In many cases the intervention was compared to usual care for an individual or an individual‐focused intervention, which meant the impact of the couplebased content could not be isolated. Three arm studies could determine whether any added benefits of couple‐focused interventions are due to adding the partner or specific content of couple‐focused interventions.

**Statement of contribution**

***What is already known on this subject?***

- Health behaviours and health behaviour change are more often concordant across couples than between individuals in the general population.
- Couple‐focused interventions for chronic conditions are more effective than individual interventions or usual care (Martire, Schulz, Helgeson, Small, & Saghafi, 2010).

***What does this study add?***

- Identified studies targeted a variety of health behaviours, with few studies in any one area.
- Further assessment of the effectiveness of couple‐focused versus individual interventions for those at risk is needed.
- Three‐arm study designs are needed to determine benefits of targeting couples versus couple‐focused intervention content.

## Background

Many health behaviours are concordant across couples (Meyler, Stimpson, & Peek, 2007), including dietary intake (Macario & Sorensen, 1998) and smoking (Graham & Braun, 1999; Stimpson, Masel, Rudkin, & Peek, 2006). This is partly due to assortative mating (the fact that couples with similar characteristics are more likely to marry) and mate selection, but may also reflect the influence spouses have on each other's health behaviours (Wilson, 2002). Couple concordance may explain risk factors for disease at the household level (Wilson, 2002). For example, spouses of patients with several illnesses are at increased risk of the diseases, including hypertension (Hippisley‐Cox & Pringle, 1998) and tuberculosis (Crampin *et al*., 2011). Also, health behaviour change tends to be concordant across couples. For example, in an observational study of couples attending a family health check‐up, changes in smoking, blood pressure, blood glucose and cholesterol level were correlated across couples 1 year after a cardiovascular lifestyle intervention programme (Pyke, Wood, Kinmonth, & Thompson, 1997). Further, when one partner adopts a healthier behaviour, the other is more likely to make a positive health behaviour change (Jackson, Steptoe, & Wardle, 2015).

Baucom Porter, Kirby, and Hudepohl (2012) characterize couple‐based interventions as either treating one partner as a coach, who assists the at‐risk partner in making health behaviour change, or focusing equally on both partners and the ways in which communication affects their health and behaviours. This framework can be used in an attempt to understand processes by which couple‐based health behaviour change interventions might work, and why and how health behaviour change interventions may be more effective for couples than individuals. Keefe *et al*. (1996), in an intervention for patients with osteoarthritis, found that while a partner‐assisted intervention lead to better long‐term adjustment for those who were more happily married, an individual intervention led to worse long‐term adjustment for those who were happily married, suggesting the value of involving the spouse in interventions. Related to this, Umberson's (1992) argument that many spouses monitor and attempt to control their spouse's health behaviours suggests that interventions that do not involve the controlling spouse are less likely to be effective. Alternatively, Lewis *et al*. (2006) developed the interdependence model of couple interaction, which proposes that partner influences are helpful when initiating health behaviour change. According to this model, couple‐focused health behaviour change interventions should therefore facilitate greater intentions to change and greater behaviour change on the part of the partner, by increasing a relational perspective on the health behaviour change (which would result in attempts to discuss behavioural change and support and influence the other partner to make behaviour changes). Also, Bandura's social cognitive theory (Bandura, 1986) suggests that reproduction of a behaviour is influenced by the environment, such that appropriate support can enhance self‐efficacy to perform a behaviour. Applying this to couple‐based interventions would suggest that support from the spouse could facilitate health behaviour change.

Evidence suggests that couple‐focused interventions may be more effective than individual interventions in facilitating long‐term maintenance of behavioural changes in one or both members of a couple (Martire & Schulz, 2007), and are more effective than either individually focused interventions or usual care for a variety of chronic conditions (Martire, Schulz, Helgeson, Small, & Saghafi, 2010). A review of weight loss interventions for couples revealed that the couple‐focused interventions led to more weight loss than stand‐alone programmes post‐intervention, but these improvements were not sustained over longer periods (Black, Gleser, & Kooyers, 1990). However, this review addressed only interventions targeting diet and exercise behaviours. Also, details of intervention content were not reported (this study was published in 1990, before reporting guidelines had been published for randomized controlled trials; Moher, Schulz, & Altman, 2001). This is important as Lewis *et al*. (2006) propose that interventions that attempt to transform motivation for behaviour change to ascribe meaning for relationships should be more successful than interventions where meaning for change is ascribed to the individual. Recent reviews (e.g., Martire *et al*., 2010) have not addressed people at risk of chronic physical illness, only those who are already managing chronic illness. However, motivation for making lifestyle changes may well be lower in individuals who are at risk of a chronic illness relative to those who have been diagnosed with one, meaning that partners may be able to play a greater role in facilitating behaviour change. Also, when an individual is diagnosed with a chronic illness, their partner often has to take on the role of carer, changing the dynamics of couple interaction (e.g., Martire *et al*., 2010). Further, in many couple‐focused intervention study designs to date, the intervention has been compared only to usual care. This means it is often unclear whether the effectiveness of such interventions is due to the behaviour change techniques used or because the interventions are couple based. Also, many studies provide individual interventions to couples, without introducing ways in which the couple can support each other and enhance the effectiveness of the intervention.

We aimed to systematically review the findings of randomized trials and non‐randomized intervention studies evaluating couple‐focused interventions for health behaviour change in populations at risk of chronic physical illness. Secondary aims were to (1) assess the design of each study and whether it isolated the couple‐based component of the intervention and (2) identify successful components of couple‐focused interventions.

## Methods

### Procedure

**Figure 1:**
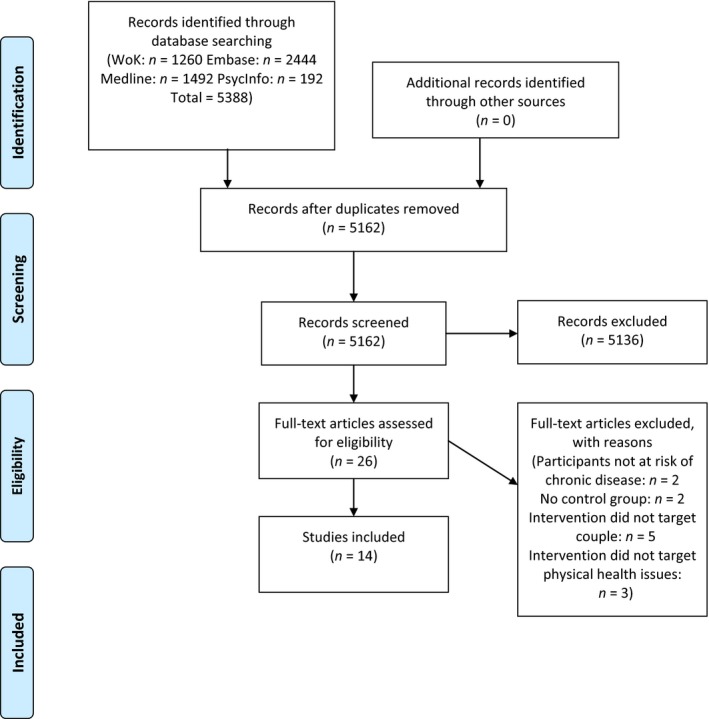
Flowchart detailing the search process.

Two methods were used to locate relevant studies: a keyword search and a backward search. Using the keyword search method, we searched the databases MEDLINE, Embase, Web of Knowledge, and PsycINFO for articles published in the English language between January 1990 (when the review on weight loss interventions (Black *et al*., 1990) was carried out, as based on a search of earlier literature, no couple‐focused interventions on other topics were identified prior to this date) and June 2014. To avoid exacerbating publication bias, we decided not to include unpublished data and dissertations (Ferguson & Brannick, 2012). Couple‐focused interventions for HIV prevention were not included as a recent review had been conducted on this topic (Burton, Darbes, & Operario, 2010). Searches included the following terms specific to couples (couple, spouse, partner, significant others, interpersonal relations) and the following terms specific to health behaviour change, which were generated by brainstorming among the authors and checked with experts in the field of health behaviour change (health behaviour, health promotion, physical activity, diets, aerobic exercise, lifestyle, self‐examination [medical], cancer screening, smoking cessation). Database‐specific strategies were created to accommodate different methods of truncation and MeSH terms. After each term had been entered into the keyword function, the couple‐related terms were combined using the OR function, and so were the health behaviour change terms. The results of the previous searches were then combined using the AND function. This generated 192 articles from PsycINFO, 1,260 from Web of Knowledge, 2,444 from Embase, and 1,492 from MEDLINE. The titles and abstracts of these articles were scanned for inclusion in the review. Overall, the keyword search yielded 26 articles. Details of the search strategy are reported in Figure 1, and the full search strategy for Web of Knowledge is reported here: (COUPLE* OR SPOUSE* OR PARTNER* OR ‘SIGNIFICANT OTHER’ OR ‘INTERPERSONAL RELATIONS’*) AND (‘HEALTH BEHAVIOR’ OR ‘HEALTH PROMOTION’ OR ‘PHYSICAL ACTIVITY’ OR ‘DIET’ OR ‘AEROBIC EXERCISE’ OR ‘LIFESTYLE’ OR ‘SELF‐EXAMINATION’ OR ‘WEIGHT LOSS’ OR ‘CANCER SCREENING’ OR ‘SMOKING CESSATION’). Some terms differed between databases. For example, the MESH term ‘self‐examination (medical)’ came up in PsycINFO, MEDLINE and Embase (which could be searched through the same platform) but not Web of Knowledge. Also, we excluded the term ‘interpersonal relations’ from Embase, as it increased the number of articles from 452 to 2,444 without identifying further articles for inclusion.

Following the keyword search, we carried out a backward search, in which we located papers by examining the reference lists of all papers identified from the first step (Meyler *et al*., 2007). This did not identify any further articles meeting the criteria.

Included studies had to: (1) include populations where at least one partner was at risk of a chronic physical illness they had not already experienced, (2) involve active participation of both partners, (3) include adults aged 18, and (4) have a control group. Studies were excluded if (1) the participants were not at risk of chronic physical illness, (2) there was no control group, and (3) the intervention did not target the couple. Both authors screened identified articles, and any discrepancies were resolved by discussion.

The following information was extracted from each study: aims, design, sample size, intervention given to partners, intervention given to control group (if applicable), length of follow‐up, measures, and findings. Details of included studies are reported in Table 1.

**Table 1:** Characteristics of included studies

Ref no	Article ref	Country	Aims	Design	Sample size (per group)	Population	Partner intervention	Control group	Length of follow‐up	Outcomes	Findings
1	Benyamini *et al*. (2011)	Israel	Determine independent effect of adding spouse involvement to a breast self‐examination (BSE) programme	RCT	140 (70)	Married women aged 24–55 (women have a 1/9 lifetime risk of developing breast cancer)	Instructions to form action plan for BSE; info for husband, who was invited to help wife fill in action plan	Instructions to form action plan for BSE	3 months 65 completed intervention, 62 in control (others only baseline measures)	*Primary*: Rates of BSE performance *Secondary*: Husbands knowledge Husbands’ involvement in wives’ health behaviours Perception of spouse support	1. Significant main effect of time on BSE performance 2. No group differences in BSE 3. Husbands who were involved more likely to report knowledge of wives’ BSE performance 4. Husbands more likely to encourage wives in experimental group 5. Women benefited most if husbands not involved in health behaviours before study
2	Burke *et al*. (1999)	Australia	Determine acceptability of, compliance with and responses to health promotion programme for couples	RCT (pilot)	39 couples	Couples who had been married/cohabiting <2 years (this period is associated with weight gain and physical inactivity, leading to obesity)	16‐week programme: nutrition, physical activity, healthy lifestyle High: modules every 2 weeks, half by mail, half at sessions Low: 1 session, then mailed every 2nd week	Offered programme after study	16 weeks following start of study (34–17/group) completed study	*Primary*: Blood pressure Heart rate Dietary intake Alcohol intake Physical activity	1. Decrease in consumption of takeaways, increase in reduced fat foods, fruit, and vegetables in intervention group relative to controls 2. Greater increase in physical activity in intervention group, but NS 3. Fall in cholesterol in intervention group relative to controls
3	Burke *et al*. (2003)	Australia	Determine longer term effect and cost‐effectiveness of health promotion programme for newly cohabiting couples	RCT	137 (47 – high‐level intervention; 47 low‐level intervention; 43 control)	Couples who had been married/cohabiting <2 years Risk as in Burke *et al*. (1999)	As Burke *et al*. (1999)	Usual care	1 year (107 completed at end of programme; 78 attended 12‐month follow‐up)	*Primary*: Dietary intake Physical activity Alcohol intake	1. Reduction in fat and saturated fat intake in high‐level intervention group relative to control, at end of intervention and 1‐year follow‐up 2. Improvement in fitness in high‐level group relative to other groups 3. Fall in total cholesterol and LDL cholesterol in high‐level group relative to control group 4. Participants in high‐level group less likely to become overweight or obese
4	Cohen *et al*. (1991)	USA	Evaluate effects of social support and home urine monitoring on success with dietary sodium reduction	RCT	107 (4 groups)	Patients being treated for essential hypertension (At risk of CHD)	*Active partner*: Patient and partner received three dietary sessions, expected to follow dietary restrictions and collect 24‐hr urine samples *Immediate feedback*: learned to test urine 2 weeks after instruction *Delayed feedback*: 18 weeks	*Passive partner*: Partner attended sessions but not involved, asked to follow diet, or collect urine samples	30 weeks (97 completed: 90.6% retention)	Urinary excretion of sodium	1. Participants able to reduce sodium intake by 50%+ 2. No effect of intervention on sodium intake 3. Patients and partners had significant correlation in urinary excretion of sodium at baseline, 6 and 18 weeks
5	Gellert *et al*. (2011)	Germany	Examine effects of social integration and exercise‐specific social support on physical activity	Quasi‐experiment	420	Men and women aged over 60 (this age group are at increased risk of CHD, cancer)	Leaflet prompting planning and self‐efficacy for physical activity, received by post	None – comparison between participating partner, non‐participating partner and single	4 weeks (343: 82% of baseline)	*Primary*: Physical activity *Secondary*: Social support	1. Greater physical activity and social support among individuals whose partners took part 2. Participants whose partners took part had more substantial increase in physical activity levels 3. No difference between singles and participants with a partner who did not participate 4. Participants who received more social support more physically active when partners took part, but other participants less physically active if received more social support
6	Lee *et al*. (2014)	USA	Assess efficacy of Korean Immigrants and Mammography–Culture‐Specific Health Intervention (KIM‐CHI), an educational program designed to improve mammography uptake among Korean American (KA) women	RCT	428 KA couples (KIM‐CHI: 217; Attention control: 211)	KA couples where the woman had not had a mammogram in past year –this group has low uptake of mammograms At average risk of breast cancer	30‐min Korean language DVD on breast cancer screening, followed by group discussion and couple‐based discussion activity at home	Couple‐focused information about improving diet	15 months (395 couples followed up)	Mammogram uptake	KIM‐CHI group were significantly more likely to attend for mammograms than control group at 6 months (*p* < .001) and 15 months (*p* = .004)
7	Manne *et al*. (2013)	USA	1. Improve colorectal cancer screening (CRCS) intentions by increasing frequency of couples’ discussions, increasing each partners’ support for other partner to have CRCS, increasing couples’ relational perspective on CRCS 2. Evaluate impact of intervention on partners’ knowledge of CRC and ind attitudes about CRCS	RCT	168 couples (GP: 80; CTP: 86)	Married couples at average risk of CRC and non‐adherent to standard CRC screening recommendations	Couple‐tailored print (CTP) booklet about CRC screening, plus Centre for Disease Control (CDC) booklet	CDC booklet	6 months (138 couples followed up)	*Primary*: CRC Screening status; Screening intentions *Secondary*: Relationship factors; Relational perspective; Support for spouse screening Discussions with spouse about CRS Knowledge and attitudes Perceived risk Benefits and barriers of screening	1. No main effect of condition on screening status (11.6% uptake) 2. Increase in intention from T1 to T2 in CTP condition 3. Increase in relationship perspective over time in CTP, for men only 4. CTP: significantly increase from husbands over time in support for wives’ CRCS 5. Women greater increase in perceived benefits after CRC than men in CTP condition (Note: Couple treated as a unit)
8	McBride *et al*. (2004)	USA	Evaluate whether training in optimal support behaviours and giving support to partners increases smoking abstinence rates among pregnant women during and after pregnancy, relative to usual care, and women‐only intervention	RCT	583 (UC: 198; Woman only 192, Partner assisted 193)	Women receiving prenatal care at a medical centre, at risk of adverse pregnancy outcomes and danger to foetus due to smoking	Late pregnancy relapse prevention kit, six counselling calls (three in pregnancy, three post‐partum), + PA adjunct describing how partner could be coach. Booklet, video included, support behaviours reinforced in counselling calls	Usual care	28 weeks pregnant; 2, 6 and 12‐months post‐partum *Follow‐up*: 28 weeks: 81% 2 months: 77% 6 months: 79% 12 months: 76%	*Primary*: Self‐reported smoking status *Secondary*: Smoking‐specific support General support	1. No differences by condition in sustained or point prevalence abstinence 2. No differences in post‐partum relapse 3. Decline in positive partner support from baseline to 12 months 4. More partners abstinent at 28 weeks in PA than UC condition
9	Øien *et al*. (2008)	Norway	Investigate parental smoking behaviour in pregnancy after introduction of prenatal, smoking cessation in primary care	NRI	Control – 1,788 Intervention – 2,051	Pregnant women who smoked (invited to bring partners), at risk of adverse pregnancy outcomes and danger to foetus	Brief intervention on diet, indoor dampness and smoking cessation	No treatment	6 weeks post‐natal: Intervention: 1,109; ctrl: 1,023	Self‐reported smoking behaviour 6 weeks post‐natal	No effect on parental smoking
10	Park *et al*. (2009)	South Korea	Examine effects of cognition‐oriented BSE intervention for women with no prior BSE experience who avoid thinking about or performing BSE and spouses	NRI	48 couples (24/group)	Married couples with no experience of breast cancer – women have a one in nine risk of developing breast cancer in their lifetime	1.5‐hr lecture, with opportunity to practice BSE while being videotaped, receive feedback on video	Lecture on breast cancer and BSE	6 months (Follow‐up rate not reported)	*Primary*: Stage of BSE *Secondary*: Knowledge of breast cancer and BSE Spousal encouragement for compliance Perceived confidence, benefits, barriers	1. Change in knowledge of BSE and breast cancer greater in experimental group 2. Greater increase in perceived confidence in experimental group 3. Significantly greater change in stage of BSE in experimental group (but no group difference)
11	Robinson *et al*. (2007)	USA	Examine role of partner assistance in learning and implementation of intervention designed to promote skin self‐examination (SSE)	RCT	130 (65/group)	Participants diagnosed with cutaneous melanoma, seen annually by physicians At risk of developing melanoma	Dyadic learning: couple‐based skills training for SSE, provision of enabling kit	Same, but solo learning	4 months (100% follow‐up)	*Primary*: Performance of SSE *Secondary*: Skills quiz Skin cancer risk Perceived risk Perceived benefit of SSE Self‐efficacy of performing SSE Skin cancer knowledge Attitudes to SSE	1. Dyadic learners believed significantly more important to perform SSE, and have a partner assist 2. Dyadic learners had significantly higher self‐efficacy for performing SSE 3. Dyadic learners showed significantly stronger intentions to perform SSE on face and skin in general 4. Significantly more dyadic learners checked skin at 4 months 5. Dyadic learning significantly more likely to review SSE guidelines, examine skin with and without a partner
12	van Jaarsveld *et al*. (2006)	UK	Examine influence of marital status and inviting both partners together on attendance at colorectal cancer screening	Retrospective analysis of trial data	4,130 adults aged 55–64	Adults aged 55–64 who had been invited for colorectal cancer screening in age group at increased risk for CRC	Both partners invited	Invited alone	Period of trial (N/A)	Attendance at colorectal cancer screening	1. Married (or cohabiting) individuals significantly more likely to attend for screening 2. Inviting partners together significantly increased attendance at screening
13	Voils *et al*. (2013)	USA	Determine effectiveness of Couples Partnering for Lipid Enhancing Strategies CouPLES on adherence to cholesterol‐lowering regime	RCT	255 (127 – intervention)	Outpatients with low‐density lipoprotein cholesterol (LDL‐C) > 76 mg/dl) (At risk of CHD)	CouPLES: 9 monthly goal‐setting telephone calls delivered by research nurse (first patients, then spouses 1 week later)	Usual care	11 months 83% follow‐up; 106/group)	Primary: Patient LDL‐C	1. No significant difference in mean LDL‐C between intervention and UC at 11 months 2. No difference in odds of meeting goal LDL‐C 3. Reduced caloric intake in intervention group, total and saturated fat intake, and percentage of calories from fat
14	Wing *et al*. (1991)	USA	Test effectiveness of family‐based approach for obese patients with Type II diabetes	RCT	49 (Together: 24; Alone: 25)	Obese patients with diabetes (At risk of CHD)	Together: participated with spouses in behavioural weight control program	Alone: participated alone	After 20‐week program; 1 year (43 patients; 42 spouses completed)	*Primary*: Weight BMI *Secondary*: HbA1c Fasting blood sugar Exercise	1. Significant weight loss and short‐term improvements in glycaemic control, reductions in fat intake, increases in exercise 2. Men did better when treated alone; women did better when treated in the ‘together’ condition 3. Spouses lost significantly more weight in ‘together’ condition 4. Correlations between patients and spouses: changes in fat intake in ‘alone’ condition, changes in exercise in both conditions

Randomized controlled trials (RCTs) and non‐randomized intervention studies were assessed using the Cochrane Collaboration Risk of Bias tool (Higgins *et al*., 2011) by both authors (EAC and NM), and any disagreements resolved by discussion. Details are reported in Table 2.

**Table 2:** Risk of bias in included studies

Study	Type of study	Was allocation sequence adequately generated?	Was allocation adequately concealed?	Was knowledge of allocated intervention adequately prevented during the study?	Were incomplete outcome data adequately addressed?	Are reports of the study free of suggestion of selective outcome reporting?	Was the study apparently free of other problems that could put it at high risk of bias?	Overall risk of bias
Benyamini *et al*. (2011)	RCT	Unclear	Yes	Unclear	No	Unclear	Yes	High
Burke *et al*. (1999)	RCT	Unclear	Unclear	Unclear	Yes	Unclear	Unclear	Unclear
Burke *et al*. (2003)	RCT	Yes	Yes	Unclear	Unclear	Unclear	Unclear	Unclear
Cohen *et al*. (1991)	RCT	Unclear	Unclear	No	Unclear	Unclear	Unclear	High
Lee *et al*. (2014)	RCT	Unclear	Unclear	No	Yes	Unclear	Unclear	High
Manne *et al*. (2013)	RCT	Unclear	Unclear	Unclear	Yes	Unclear	Unclear	Unclear
McBride *et al*. (2004)	RCT	Unclear	Unclear	Unclear	Yes	Unclear	Unclear	Unclear
Oien *et al*. (2008)	Prospective cohort study	No	No	No	No	Unclear	No	High
Park *et al*. (2009)	Time‐series non‐equivalent control group	Unclear	Unclear	Unclear	Unclear	Unclear	No	High
Robinson *et al*. (2007)	RCT	Unclear	Unclear	Unclear	Yes	Unclear	Yes	Unclear
Voils *et al*. (2013)	RCT	Yes	Yes	Unclear	Unclear	Yes	Yes	Unclear
Wing *et al*. (1991)	RCT	Unclear	Unclear	Unclear	Yes	Unclear	Unclear	Unclear

## Results

On reading, 12 of the 26 studies were excluded. Two targeted healthy adults who were not at risk of a specific chronic illness (Niederhauser, Maddock, LeDoux, & Arnold, 2005; Wallace, Raglin, & Jastremski, 1995), two had no control group (Homan, Litt, & Norman, 2012; Shoham, Rohrbaugh, Trost, & Muramoto, 2006), two targeted the at‐risk individuals through their female partners (Chan, Leung, Wong, & Lam, 2008; Matsuo *et al*., 2010), partner inclusion was not compulsory in three (de Vries, Bakker, Mullen, & van Breukelen, 2006; Prestwich *et al*., 2005; Wakefield & Jones, 1998), and three did not target physical health issues (Fisher, Wynter, & Rowe, 2010; Midmer, Wilson, & Cummings, 1995; Sciacca, Dube, Phipps, & Ratliff, 1995).

Overall, 14 studies carried out by 13 research groups were included in this review. The sample size ranged from 39 couples (Burke *et al*., 1999) to 3,839 (Øien, Storrø, Jenssen, & Johnsen, 2008). The studies were carried out in the USA (Cohen *et al*., 1991; Lee *et al*., 2014; Manne *et al*., 2013; McBride *et al*., 2004; Robinson, Turrisi, & Stapleton, 2007; Voils *et al*., 2013; Wing, Marcus, Epstein, & Jawad, 1991), Australia (Burke, Giangiulio, Gillam, Beilin, & Houghton, 2003; Burke *et al*., 1999), the United Kingdom (van Jaarsveld, Miles, Edwards, & Wardle, 2006), Israel (Benyamini, Ashery, & Shiloh, 2011), South Korea (Park, Song, Hur, & Kim, 2009), Germany (Gellert, Ziegelmann, Warner, & Schwarzer, 2011), and Norway (Øien *et al*., 2008).

The studies targeted the following health behaviours: colorectal cancer screening, breast self‐examination (BSE), skin self‐examination, obesity, diet, smoking in pregnancy, and physical activity.

There were ten RCTs, two non‐randomized intervention studies, and two studies in which trial data were retrospectively analysed. Six studies utilized a usual care/no‐treatment control group. Four of the 10 RCTs compared a couple‐based intervention to an intervention targeting the individual. One non‐randomized study compared people joining exercise programmes as couples relative to individuals. One study retrospectively used trial data to compare the effect of inviting individuals versus both members of a couple to colorectal cancer screening, one RCT compared varying levels of partner involvement, one non‐randomized intervention study compared two couple‐focused interventions differing in intensity, and one RCT compared two interventions targeting the couple.

The studies targeted a variety of populations (both men and women unless otherwise specified). Populations included individuals at average risk of colorectal cancer, couples where the woman had never had breast cancer (which one in eight women will experience in their lifetime; Cancer Research UK, 2014), individuals who had not had a mammogram within the past year, individuals who had been married or cohabiting for <2 years (this period is often associated with weight gain and physical inactivity), obese individuals with diabetes, adults being treated for essential hypertension, individuals with low‐density lipoprotein (LDL) cholesterol > 76 mg/dl being treated in primary care, persons at risk of melanoma, and couples aged over 60, who are at greater risk of chronic illness than the general population.

Behaviour change outcomes included both objective measures (e.g., attendance at screening, cholesterol levels, systolic and diastolic blood pressure, heart rate, weight, hip and waist circumference) and self‐report measures, including self‐reported levels of physical activity, diet, self‐examination, and smoking. The interventions varied considerably in intensity, from an invitation to screening (van Jaarsveld *et al*., 2006), to a 16‐week programme focusing on health behaviours (Burke *et al*., 1999, 2003). Length of follow‐up varied from a single visit post‐intervention where measurements were taken (e.g., blood pressure, heart rate; Burke *et al*., 1999) to 15 months (Lee *et al*., 2014). This information is reported in Table 1.

Due to the diverse nature of the interventions and variety of populations studied, it was not possible to do a meta‐analysis. Studies differed considerably regarding length of follow‐up, intervention content, and outcome measure, meaning that no direct comparisons could be made. Martire *et al*. (2010) were able to conduct a meta‐analysis because they had ‘three outcomes for which there were an adequate number of effect sizes (defined as at least 8–10) for aggregation’. However, this was not the case for the studies included in this review. Although couple‐focused interventions have been carried out to address health behaviour change in individuals at risk of chronic physical illness, few have been carried out in one area. Effect sizes have been reported where it was possible to calculate them. In some cases, insufficient information was reported to enable the calculation of effect sizes.

### Content of interventions

Only three interventions reported using couple‐based behaviour change techniques (e.g., getting the spouse to focus on patient goals). Manne *et al*. (2013) provided couples with a couple‐tailored booklet, based on responses members of the couple had given to a survey (which included responses to barriers). This booklet contained pictures of couples, explained the importance of including the spouse in the screening decision, and described ways to have a positive discussion about screening. The invitation letter to the study asked the participant to read the booklet and discuss it with their spouse. In Voils *et al*. (2013), the intervention consisted of nine monthly goal‐setting calls, which were made to patients and spouses separately. Initially, education on diet and self‐management was provided to both patients and spouses, and spouses were provided with orientation to support patient goal achievement (focusing on patients’ goals) and asked to generate a specific behaviour plan they would follow to support patient goal achievement. In the second telephone call, patients were required to set goals and create action plans. Spouses were informed about these goals and action plans, and received suggestions on how to help patients. In subsequent months, while patients monitored their progress, spouses were informed of changes and continued to receive suggestions to support patients. Finally, in Wing *et al*. (1991), couples participated in a 20‐week behavioural weight control programme with 12 weekly sessions and four bi‐weekly sessions. The treatment programme emphasized the importance of spouse support for modifying diet and exercise. Couples were taught to identify things their spouse could do to help them comply with the programme, and required to make a contract to provide at least one form of practical support per week. Spouses were taught listening skills and to praise each other for appropriate changes in behaviour. Couples were taught to identify joint problems and work together to develop solutions.

### Results of the quality assessment

Randomized trials and non‐randomized intervention studies are addressed separately. Most RCTs (8/10) were classified as having unclear risk of bias overall, but three were classified as having high risk of bias for one of the key domains, and therefore high risk of bias overall. Most trials were classified as having unclear risk of bias because they had not reported how the allocation sequence was generated and concealed, or whether blinding was accurate during the study. Six of 10 RCTs addressed incomplete data adequately, one did not, and in three, it was unclear. In nine of 10 RCTs, it was not clear whether outcomes were reported selectively; only one of the RCTs had published a study protocol.

Two non‐randomized intervention studies (Øien *et al*., 2008; Park *et al*., 2009) were assessed according to the Risk of Bias tool. These studies were assessed as having high risk of bias. In non‐randomized trials, even when the experimental and control groups appear comparable at baseline, the effect size is at risk of bias due to residual confounding (Reeves, Deeks, Higgins, & Wells, 2008). In one of the two non‐randomized intervention studies, the allocation sequence was not adequately generated or concealed (recruitment to the intervention and control groups took place over separate time periods). In one study, it was clear that blinding had not occurred. None addressed incomplete outcome data adequately. In both, it was unclear whether the outcomes had been reported selectively. The two studies based on retrospective analysis of trial data (Gellert *et al*., 2011; van Jaarsveld *et al*., 2006) could not be assessed for risk of bias without reference to the original trial papers.

Only six of the 14 studies reported carrying out a power calculation (Benyamini *et al*., 2011; Burke *et al*., 1999, 2003; Lee *et al*., 2014; Park *et al*., 2009; Voils *et al*., 2013). Some of the remaining studies may have been underpowered (e.g., Wing *et al*., 1991). However, insufficient detail was given of statistical assumptions made when calculating sample size.

### Summary of study findings

#### Attendance at cancer screening

Retrospective analysis of trial data revealed that individuals were more likely to attend for colorectal cancer screening following two invitations by post if they were part of a couple where both members were invited (OR = 1.34; 95% CI 1.14–1.58; van Jaarsveld *et al*., 2006). Similarly, an RCT of a couple‐based educational programme about breast screening for Korean Americans who had not had a mammogram in the past year led to increased uptake of mammograms at 6 months (*p* < .001) and 15 months (*p* = .004) relative to a couple‐based educational programme about having a healthy diet (Lee *et al*., 2014). However, an RCT targeting couples where both members were non‐adherent with colorectal cancer screening recommendations demonstrated no difference in uptake of colorectal cancer screening in individuals following receipt of a couple‐tailored booklet versus an individually focused booklet (Manne *et al*., 2013).

#### Performance of cancer screening

An RCT showed that instructions to perform an action plan for BSE with a partner was no more effective than instructions to perform the same action plan alone (Benyamini *et al*., 2011). Similarly, an intervention comparing a lecture on BSE alone versus a lecture plus the opportunity to be videotaped carrying out BSE and receive feedback on performance (both couple‐focused) demonstrated no group differences in performance of BSE, or knowledge about BSE and breast cancer (Park *et al*., 2009). However, participants in a couple‐focused skin self‐examination programme (10 min training in skin self‐examination plus skills training) were significantly more likely to check their skin 4 months post‐intervention (64.6% vs. 30.8%; *p *<* *.001), and had significantly greater self‐efficacy for skin self‐examination than those taught the same techniques alone (Robinson *et al*., 2007).

#### Smoking in pregnancy

A non‐randomized intervention study (Øien *et al*., 2008) of 3 min of advice given to expectant couples by a health care professional during an antenatal appointment did not influence smoking cessation 6 weeks post‐birth. Similarly, an RCT of a couple‐based intervention (six counselling calls; three during pregnancy, three post‐partum) supplemented by a booklet and video did not increase smoking cessation at 12 months post‐partum relative to usual care (McBride *et al*., 2004).

#### Physical activity

A non‐randomized intervention study found adults aged over 60 were more likely to remain in an exercise programme at 4‐week follow‐up if their partners also participated than if they did not participate (Cohen's *D* = 0.46, 95% CI 0.14–0.78; Gellert *et al*., 2011).

#### Nutrition/weight control

An RCT of a couple‐based intervention consisting of nine monthly goal‐setting telephone calls to individuals with high cholesterol levels and support planning calls to spouses compared to usual care (clinical management by providers) showed no effect on LDL cholesterol levels at 11 months follow‐up (Voils *et al*., 2013). Similarly, an RCT comparing an intervention targeted at individuals with essential hypertension and their partners where both partners were active participants (attended three dietary lessons 2 weeks apart, followed dietary restrictions and collected 24‐hr urine samples), to an intervention where the non‐hypertensive partner was a ‘passive participant’ (attended the dietary lessons only) did not lead to group differences (Cohen *et al*., 1991). However, in an RCT of a weight control programme for obese individuals with type II diabetes comparing individuals treated alone and with a partner (Wing *et al*., 2011) obese women lost more weight when treated with a partner, whereas obese men lost more weight when treated alone, *F*(1,* *38) = 7.7, *p < *.01 (Wing *et al*., 1991). Spouses lost more weight when treated together than alone (Cohen's *D* = 1.52, 95% CI 0.89–2.16).

#### Multiple health behaviours

A pilot study of a 16‐week programme on nutrition and physical activity for couples who had been married or cohabiting <2 years led to a reduction in total fat consumption (*p* = .04), saturated fat intake (*p* = .01), and cholesterol levels (*p* = .02) (Burke *et al*., 1999). A larger scale RCT of the same 16‐week programme led to a reduction in fat consumption (*p* = .01), overall cholesterol levels (*p* = .02), and LDL cholesterol levels (*p* = .02) (Burke *et al*., 2003). However, no primary outcomes were named, and insufficient information was provided to enable calculation of effect sizes.

## Discussion

We carried out a systematic review of RCTs and non‐randomized intervention studies evaluating couple‐focused interventions for health behaviour change in populations at risk of chronic physical illness. The studies we identified targeted a variety of outcomes and behaviours, with few studies in any one area. Interventions for couples led to improvements in attendance at cancer screening, skin self‐examination, increased breastfeeding, reduction in dietary fat intake, weight loss, and increased exercise. However, they did not increase smoking cessation or BSE.

Two retrospective analyses of intervention studies showed individuals were more likely to participate in health behaviours with a partner than alone (Gellert *et al*., 2011; van Jaarsveld *et al*., 2006). Two trials (Burke *et al*., 1999, 2003) of five comparing couple‐focused interventions to usual care showed couple‐focused interventions were significantly more effective than usual care in improving health outcomes for couples, and the other three (McBride *et al*., 2004; Øien *et al*., 2008; Voils *et al*., 2013) found no effect of couple‐based interventions relative to usual care. Similarly, none of the three studies using couple‐based behaviour change techniques demonstrated a significant result. Based on these eight interventions, it is unclear whether targeting couples will improve the effectiveness of health behaviour change interventions.

Evidence for the effectiveness of couple‐focused interventions relative to individual interventions is more mixed, but expected given the varied targeted outcomes and intervention approaches considered. Two of four RCTs showed that couple‐based interventions were more effective than individual interventions, and two RCTs demonstrated no difference between the two. Two studies comparing couple‐based interventions differing in intensity found no differences between the two. Finally, in an RCT, a couple‐based intervention targeting the behaviour in question led to greater improvements in the health behaviour than an active couple‐based control group.

Inviting both members of a couple to colorectal cancer screening led to increased attendance at screening relative to inviting only one member. Such a low‐cost intervention could easily be implemented in the UK health care system, and may also be relevant to other health screening programmes that are applicable to both sexes.

The studies differed considerably with regard to population, type of intervention, outcome measures, length of follow‐up, and part of the world they were carried out in. This meant it was not possible to carry out a meta‐analysis. The two areas that previously have had a number of couple‐focused intervention studies conducted, weight loss and HIV prevention, found that couple‐focused interventions were successful in enabling weight loss post‐treatment (Black *et al*., 1990), although the effects were not sustained, and reduced unprotected sexual intercourse and increased condom use relative to control groups (Burton *et al*., 2010). This evidence for possible effectiveness of couple‐based interventions suggests that, in the health behaviour change areas identified in our review, further studies are needed to assess the effectiveness of couple‐based versus individual interventions, despite increased resources and logistical challenges involved with trying to recruit and retain couples (Coyne & Lepore, 2006; McGrath *et al*., 2010).

No studies were classified as having low risk of bias. Three RCTs and two non‐randomized intervention studies were classified as having a high risk of bias. In most cases, the requirements of the Cochrane collaboration risk of bias tool were not met due to unclear reporting, partly because many were carried out pre‐CONSORT guidelines. However, it is important to note that in one of the non‐randomized studies (Park *et al*., 2009), there was no option to take part as an individual rather than a couple. Further, only six studies reported sample size or power calculations and few discussed potential bias in their results. A limitation of the review is that we focused only on peer‐reviewed published studies and may have missed relevant studies from the grey literature. However, it is unlikely that studies from the grey literature would have been better quality than the studies in our review, as poorer quality studies are less likely to be published in peer‐reviewed journals.

Martire *et al*. (2010) carried out a review of couple‐related interventions for chronic illness. Martire's review identified similar concerns regarding the design of couple‐focused intervention and highlighted three main design and measurement issues that researchers should consider in testing such interventions: (1) that researchers’ reference research and theory that led them to use couple‐based interventions, (2) that outcomes are assessed for the partner as well as the patient, and (3) that couple‐ and individual‐oriented approaches be compared. Their review significantly differs from this review as the interventions they assessed looked mainly at influencing relationship functioning, rather than involving the spouse to provide support and facilitate behaviour change (their theoretical framework was that chronic illness is likely to lead to a change in relationship functioning between members of the couple). Nevertheless, based on this review, we agree with Martire *et al*. recommendations, and concur with their comment that improvements in methodological quality and attention to published guidelines for the reporting of clinical trials, for example the CONSORT guidelines (Moher *et al*., 2001) are required when carrying out research on couples.

We noted the following methodological issues with the studies. First, very few provided a theoretical rationale for the use of couple‐based interventions. Second, as very few studies addressed changes in couple functioning, we were unable to determine possible mechanisms for intervention effectiveness. Third, only one study used dyadic analysis. Such analysis would enable researchers to account for the correlation between patients and partners in their health behaviours, leading to increased understanding of possible actor and partner effects (Kenny, Kashy, & Cook, 2006). Fourth, many studies did not report details of intervention content. This is important, as couple‐based behaviour change techniques may be more effective than techniques targeting the individual (Lewis *et al*., 2006). Fifth, many papers did not report the necessary information to enable calculation of effect sizes. Some did not even report means and standard deviations. This is important as without this information, it is impossible to determine the effectiveness of an intervention. Finally, only four RCTs compared couple‐focused and individual interventions, and only one compared a couple‐focused intervention to a control group of couples. Three‐arm studies comparing an individual intervention, a control group of couples, and a couple‐focused intervention are required to (1) determine any added benefits of couple‐focused interventions relative to individual interventions and (2) determine whether those added benefits are due to merely adding the partner or the specific content of couple‐focused interventions.

### Conclusions

Research has demonstrated high concordance between partners’ health behaviours, and there is a sound theoretical basis for the effectiveness of couple‐focused interventions for health behaviour change. However, many of the couple‐focused intervention studies reported in the literature have important limitations. The risk of bias in all of the studies identified in this review leaves us with no studies to direct our understanding on an important topic. Further methodologically sound, rigorously reported, and analysed couple‐focused interventions are therefore required in order to determine added benefits of couple‐based interventions relative to evidence‐based individual interventions and identify mechanisms of change. Studies, ideally randomized controlled trials, are needed which publish protocols prior to starting recruitment, report details of the allocation sequence, conceal allocation, prevent knowledge of the allocated intervention during the study, and correctly address incomplete outcome data in the analysis (Higgins *et al*., 2011). For behavioural scientists to ensure their studies are rigorous enough to be taken seriously and implemented in practice, this shift to enhanced transparency in data collection and reporting is essential.

## Conflict of interest

All authors declare no conflict of interest.
